# The complete mitochondrial genome of *Hemiclepsis yangtzenensis* (Clitellata: Glossiphoniidae)

**DOI:** 10.1080/23802359.2022.2070039

**Published:** 2022-05-05

**Authors:** Ti-Lin Yi, Meng-Ting Pei, Zhi-Wei Xu, Dai-Qing Yang

**Affiliations:** a School of Animal Science, Yangtze University, Jingzhou, China; b Hubei Provincial Engineering and Technology Research Center for Monopterus albus, Jingzhou, China; c Jingzhou Institute of Technology, Jingzhou, China

**Keywords:** *Hemiclepsis yangtzenensis*, leech, mitochondrial genome, phylogenetics

## Abstract

We present the complete mitochondrial genome sequence of a recently described new leech species named *Hemiclepsis yangtzenensis* Yang & Bolotov 2021 collected in central China. The mitochondrial genome is 14,984 bp in length and consists of 13 protein-coding genes, 2 ribosomal RNA genes, and 22 transfer RNA genes, all of which are encoded on a single strand. It exhibited a strong A + T bias of 72.87%. There is a large non-coding region (614 bp) located between the *tRNA-Arg* and *tRNA-His* genes, wherein we identified 40 short dispersed repeats, 13–22 bp long, 8 of which were direct, 20 inverted, and 12 palindromic. Phylogenetic analysis of 20 Hirudinea mitogenome sequences resolved monophyletic Glossiphoniidae, and *H. yangtzenensis* formed a sister lineage with *Glossiphonia concolor.*

Leeches (Annelida: Clitellata: Hirudinea) are a specialized group (>680 species) of ectoparasites or predators that mostly inhabit freshwater habitats (Sket and Trontelj 2008). Leeches play an important role in the ecosystem, so they are used as an indicator of environmental stress (Koperski 2017). They are also used in neurobiological and developmental studies (Le Marrec-Croq et al. 2013). Due to a lack of molecular data, phylogeny and taxonomy of Hirudinea (and Annelida) remain only partially resolved (Weigert and Bleidorn 2016). Importantly, in contrast to early reports, which suggested that mitochondrial architecture is highly conserved in Annelida, with all genes encoded on a single strand; a more complex picture emerged during the last ten years, with a number of lineages exhibiting highly rearranged architecture, and some even have genes encoded on both strands (Oceguera-Figueroa et al. 2016; Weigert et al. 2016; Jiménez-Armenta et al. 2020; Daffe et al. 2021; Ye et al. 2021).

To contribute to the understanding of mitogenomic evolution in annelids, and generate data for future taxonomic and phylogenetic studies, in this study, we sequenced and characterized the complete mitochondrial genome of a very recently discovered and described new leech species *Hemiclepsis yangtzenensis* Yang & Bolotov 2021 (Rhynchobdellida: Glossiphoniidae) (Xu et al. 2021). The specimen was collected from the skin of a *Monopterus albus* host in a fish breeding pond at the Aquatic Economic Animal Research Center of Yangtze University, 30.3590°N, 112.1376°E, Jingzhou, Hubei Province, China. The animal use protocol for this study was approved by the Animal Care and Use Committee of Hubei Province (China). The host being farmed fish, and the target species an unprotected invertebrate, no additional permits were required for sampling. The type specimen is deposited in the Museum of Hydrobiological Sciences, Institute of Hydrobiology, Chinese Academy of Sciences, Wuhan, Hubei Province, China (http://english.ihb.cas.cn/rh/rd/center12/201304/t20130418_101034.html; contact person: Zhiwei-Xu; e-mail: 2692420332@qq.COM) under the voucher number 18972378353.

The mitogenome was sequenced, assembled, and annotated following the methodology outlined before (Zou et al. 2017). Briefly, primers designed to match generally conserved regions of target genes were used to amplify short fragments of *16S*, *12S*, *cox1*, *atp6*, *cytb*, and *nad5.* Specific primers were designed based on these conserved regions sequences and used to amplify the remaining mitogenome sequence in several PCR reactions. The PCR reactions were carried out with LA Taq polymerase (Supplementary Data); 35 cycles comprised 94 °C for 30 s, 50 °C 30 s, and 72 °C for 1 min per 1 kb. PCR products were sequenced using Sanger sequencing. Sequences were assembled using DNAstar (Burland 2000), whereas annotation was conducted using Geneious (Kearse et al. 2012) and adjusted manually.

The complete mitochondrial genome (GenBank No. MN106285) is 14,984 bp in length. It encodes 13 protein-coding genes, 2 ribosomal RNA genes, and 22 transfer RNA genes (Supplementary data). All genes are encoded on a single strand. It exhibited very similar architectural features to other available Glossiphoniidae species. As this is the first sequenced mitogenome for the genus *Hemiclepsis*, comparisons to congeneric species currently remain impossible. It exhibited an identical gene order to another Glossiphoniidae species, *Haementeria officinalis* (Oceguera-Figueroa et al. 2016). Overall, gene order was almost identical to most other Hirudinea mitogenomes, notwithstanding rearrangements of individual tRNA genes (Supplementary Data).

The nucleotide composition of the mitogenome was 37.66% T, 15.04% C, 35.21% A, and 12.09% G, with a strong A + T bias of 72.87%, which is similar to other annelids (Ye et al. 2021). The G + C content of protein-coding genes was 26.61% (length: 11,134 bp), tRNAs 26.79% (length: 1,407 bp), and rRNAs 53.54% (length: 1,903) (Supplementary Data).

All protein-coding genes shared the same start codon, ATG, with the exception of *cox3*, which started with TTG. Seven protein-coding genes used the TAA stop codon (*cox1*, *cox3*, *nad6*, *cytb*, *atp6*, *nad4l*, and *nad2*), *nad3* used the TAG stop codon, and five genes (*cox2*, *atp8*, *nad4*, *nad1*, and *nad5*) had an incomplete stop codon T––. Incomplete stop codons are very frequent in mitogenomes of leeches, and they are most likely completed by post-transcriptional polyadenylation (Ojala et al. 1981; Oceguera-Figueroa et al. 2016). Five short non-coding regions (NCR) ranging from 1 bp to 5 bp and one large NCR (614 bp) were identified in the mitochondrial genome of *H. yangtzenensis*. This large NCR is located between *tRNA-Arg* and *tRNA-His* genes. REPuter tool (Kurtz et al. 2001) identified 40 short dispersed repeats, 13–22 bp long, in the NCR. Among these, 8 were direct, 20 inverted, and 12 were palindromic repeats. Tandem repeats have been reported in NCRs of some other annelids (Li et al. 2015).

Phylogenetic analysis was conducted on a dataset comprising almost all available Hirudinea mitogenomes (Supplementary Data). PhyloSuite (Zhang et al. 2020) was used to standardize annotation, extract data, and conduct phylogenetic analysis using nucleotide sequences of 13 concatenated protein-coding genes with the help of several plug-in programs: genes were aligned using the codon mode, and the accurate G-INS-i strategy in MAFFT (Katoh and Standley 2013), concatenated using PhyloSuite, and phylogeny was reconstructed using IQ-tree (Trifinopoulos et al. 2016) with 10,000 ultrafast bootstraps (Minh et al. 2013) and the most parameter-rich evolutionary model GTR + G+I (Supplementary Data). iTOL was used to visualize the phylogeny (Letunic and Bork 2007). Glossiphoniidae was monophyletic, and *H. yangtzenensis* formed a sister lineage to *Glossiphonia concolor* (Figure 1).

**Figure 1.**
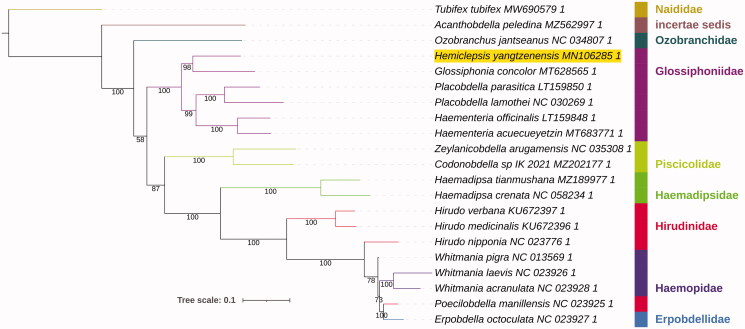
Phylogenetic tree inferred from the nucleotide sequences of 13 concatenated protein-coding genes using IQ-Tree. Bootstrap support values are shown at the nodes. Family identity is shown, *Hemiclepsis yangtzenensis* is highlighted by a yellow background, and *Tubifex tubifex* (Clitellata: Haplotaxida) is the outgroup.

## Supplementary Material

Supplemental Material

## Data Availability

The data that support the findings of this study are openly available in the GenBank of NCBI (https://www.ncbi.nlm.nih.gov/) under the accession number MN106285 (https://www.ncbi.nlm.nih.gov/nuccore/MN106285.1/).
