# Supplementary material for: The complete mitochondrial genome of Hemiclepsis yangtzenensis (Clitellata: Glossiphoniidae)
Source: Mitochondrial DNA B Resour. 2022 May 5;7(5):772–4. doi: 10.1080/23802359.2022.2070039 (PMC9090425; doi:10.1080/23802359.2022.2070039)
Supplement: Supplemental Material [file TMDN_A_2070039_SM5934.docx]

**Supplementary data for**

**The complete mitochondrial genome of *Hemiclepsis yangtzenensis* (Glossiphoniidae)**

Ti-Lin Yi, Meng-Ting Pei, Dai-Qing Yang

**Supplementary methods**

**PCR Reaction System**

TaKaRa LATaq (5 U/μl) 0.5μl

10×LATaq BufferII (Mg2+Plus) 5μl

dNTP Mixture (2.5 mM each) 8μl

Template 60ng

Primer1 0.2 -1.0 μM (final conc.)

Primer2 0.2 -1.0 μM (final conc.)

DdH_2_O up to 50μl

**Phylogenetic analysis**

In order to infer phylogenetic relationships of *H. yangtzenensis*, we retrieved all available Hirudinea mitogenomes from GenBank. After removing duplicated, unannotated, and incomplete mitogenomes, 20 mitogenomes were left in the dataset. All available Glossiphonidae mitogenomes were kept in the analysis.

A recent study has shown that the use of the most parameter-rich evolutionary model GTR+G+I allows skipping the evolutionary model selection step in phylogenetic analyses without producing any detrimental effects on the accuracy of topology (Abadi et al. 2019).

**Supplementary results**

Table S1. The base composition of the mitochondrial genome of *Hemiclepsis yangtzenensis*

|  | T(U)% | C% | A% | G% | Length(bp) | GC% | AT% |
| --- | --- | --- | --- | --- | --- | --- | --- |
| Protein coding genes | 39.03 | 15.48 | 34.35 | 11.13 | 11134 | 26.61 | 73.39 |
| tRNAs | 35.11 | 12.94 | 38.10 | 13.86 | 1407 | 26.79 | 73.21 |
| rRNAs | 33.05 | 12.72 | 40.83 | 13.40 | 1903 | 53.54 | 46.46 |
| Total | 37.66 | 15.04 | 35.21 | 12.09 | 14984 | 27.13 | 72.87 |

Table S2. The architecture of the mitochondrial genome of *Hemiclepsis yangtzenensis*

| **Gene** | **From** | **To** | **Size(bp)** | **Start Codon** | **Stop Codon** | **Anticodon** |
| --- | --- | --- | --- | --- | --- | --- |
| COX1 | 1 | 1554 | 1554 | ATG | TAA |  |
| tRNA-Asn(N) | 1538 | 1600 | 63 |  |  | GTT |
| COX2 | 1601 | 2281 | 681 | ATG | T |  |
| tRNA-Asp(D) | 2280 | 2344 | 65 |  |  | GTC |
| ATP8 | 2345 | 2503 | 159 | ATG | T |  |
| tRNA-Tyr(T) | 2502 | 2568 | 67 |  |  | GTA |
| tRNA-Gly(G) | 2567 | 2628 | 62 |  |  | TCC |
| COX3 | 2629 | 3432 | 804 | TTG | TAA |  |
| tRNA-Gln(Q) | 3407 | 3475 | 69 |  |  | TTG |
| NAD6 | 3477 | 3947 | 471 | ATG | TAA |  |
| CYTB | 3940 | 5076 | 1137 | ATG | TAA |  |
| tRNA-Trp(W) | 5075 | 5139 | 65 |  |  | TCA |
| ATP6 | 5141 | 5845 | 705 | ATG | TAA |  |
| tRNA-Arg(R) | 5847 | 5905 | 59 |  |  | TCG |
| tRNA-His(H) | 6520 | 6582 | 63 |  |  | GTG |
| NAD5 | 6583 | 8284 | 1702 | ATG | T |  |
| tRNA-Phe(H) | 8285 | 8346 | 62 |  |  | GAA |
| tRNA-Glu(E) | 8347 | 8416 | 70 |  |  | TTC |
| tRNA-Pro(P) | 8409 | 8472 | 64 |  |  | TGG |
| tRNA-Thr(T) | 8473 | 8534 | 62 |  |  | TGT |
| NAD4L | 8535 | 8828 | 294 | ATG | TAA |  |
| NAD4 | 8822 | 10156 | 1335 | ATG | T |  |
| tRNA-Cys(C) | 10155 | 10218 | 64 |  |  | GCA |
| tRNA-Met(M) | 10219 | 10280 | 62 |  |  | CAT |
| *rrnS*(12S) | 10281 | 11019 | 739 |  |  |  |
| tRNA-Val(V) | 11020 | 11083 | 64 |  |  | TAC |
| *rrnL*(16S) | 11084 | 12247 | 1164 |  |  |  |
| tRNA-Leu*(CUN)*(L1) | 12248 | 12309 | 62 |  |  | TAG |
| tRNA-Ala(A) | 12310 | 12371 | 62 |  |  | TGC |
| tRNA-Ser*(UCN)*(S2) | 12370 | 12436 | 67 |  |  | TGA |
| tRNA-Leu*(UUR)*(L2) | 12437 | 12497 | 61 |  |  | TAA |
| NAD1 | 12498 | 13433 | 936 | ATG | T |  |
| tRNA-Ile(I) | 13432 | 13493 | 62 |  |  | GAT |
| tRNA-Lys(K) | 13494 | 13557 | 64 |  |  | TTT |
| NAD3 | 13558 | 13911 | 354 | ATG | TAG |  |
| tRNA-Ser*(AGN)*(S1) | 13909 | 13976 | 68 |  |  | TCT |
| NAD2 | 13978 | 14979 | 1002 | ATG | TAA |  |


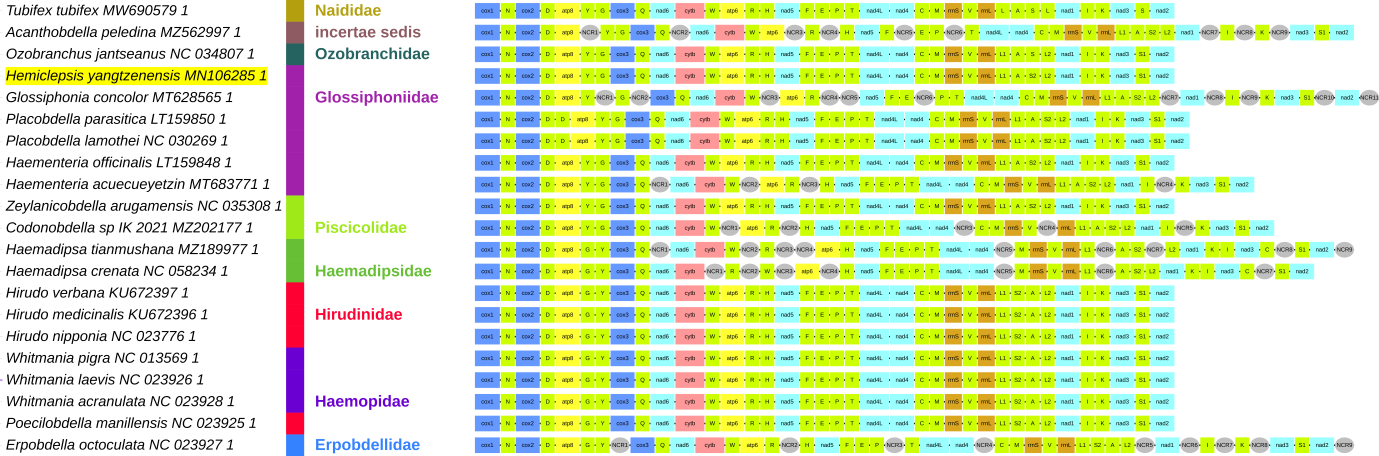


Figure S1. Gene orders in Hirudinea.
